# Towards a Guided Regeneration of Renal Tubules at a Polyester Interstitium

**DOI:** 10.3390/ma3042369

**Published:** 2010-03-26

**Authors:** Will W. Minuth, Lucia Denk, Anne Glashauser

**Affiliations:** Department of Molecular and Cellular Anatomy, University of Regensburg, University Street 31, 93053 Regensburg, Germany; E-Mails: lucia.denk@vkl.uni-regensburg.de (L.D.); anne.glashauser@vkl.uni-regensburg.de (A.G.)

**Keywords:** polyester fleece, artificial interstitium, kidney, stem/progenitor cells, regeneration, aldosterone

## Abstract

Stem/progenitor cells are promising candidates for a therapy of renal failure. However, sound knowledge about implantation and regeneration is lacking. Therefore, mechanisms leading from stem/progenitor cells into tubules are under research. Renal stem/progenitor cells were isolated from neonatal rabbit kidney and mounted between layers of polyester fleece. It creates an artificial interstitium and replaces coating by extracellular matrix proteins. Tubulogenic development is induced by aldosterone. Electron microscopy illuminates growth of tubules in close vicinity to polyester fibers. Tubules contain a differentiated epithelium. The spatial extension of tubules opens a new strategy for testing morphogenic drugs and biocompatible fleece materials.

## 1. Introduction

### 1.1. Limited regeneration of renal parenchyma

The capability of parenchyma regeneration is limited in patients with chronic or acute renal failure [1,2,3]. In view of this clinical background, the question arose as to which molecular processes hamper a diseased kidney to form new parenchyma [4,5,6]. Due to this reason, numerous investigations have been made to find therapeutic strategies promoting the controlled regeneration of renal parenchyma by drug delivery, cell implantation and tissue engineering [7,8,9,10]. An ideal form of future therapy would be to induce a process of regeneration by morphogenic factors, to guide the spatial growth of nephrons along innovative biomaterials as basal lamina substitute and to stimulate in parallel the microvascularization [4,12].

It is imaginable that the renewal of parenchyma occurs *via* stimulation of non-diseased adult kidney cells, by an implantation of stem cells or by an activation of organ-specific stem/progenitor cells [13,14,15,16,17,18]. In addition to the source of regenerating cells, the preceding developmental process to form nephron-specific segments is of enormous importance. During this step, the cells have to form spatially organized tubules containing a distinct lumen and a basal lamina [19,20]. Most problematic is that the process of regeneration does not occur in an optimal environment, but is influenced by inflammatory processes and reduced vascularization at the site of kidney damage.

Up to date sound cell biological information about promoting and hampering parameters influencing spatial tubule development are scarce. For that reason, innovative protocols for therapeutic induction and a subsequent guiding of the regeneration process are in the focus of intense research.

### 1.2. Sensitivity of renal cells

A process of regeneration can only be influenced when exact information about determination, proliferation, polarization and functional differentiation of renal cells is available. To obtain insights in their physiological reactions, multiple culture experiments with renal epithelial cells were performed.

**Figure 1:**
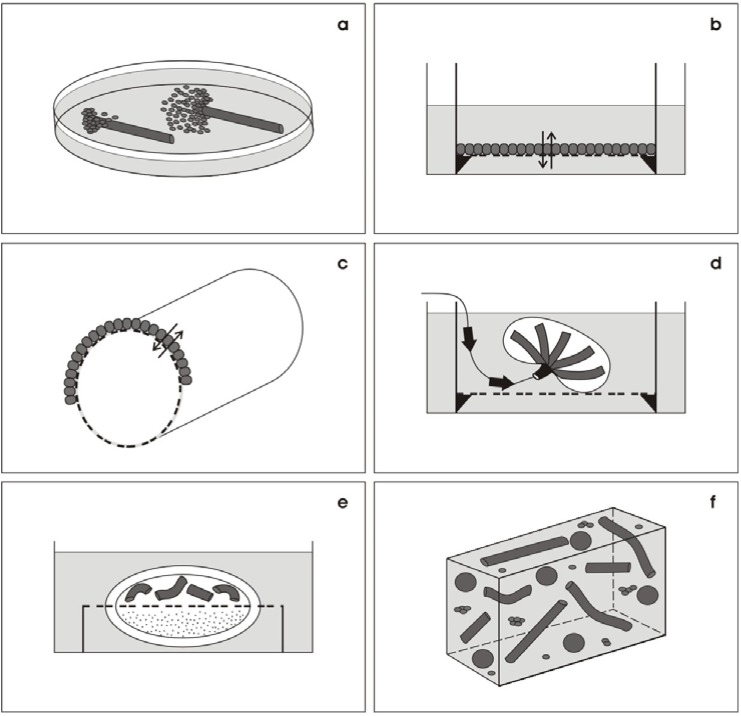
(a) Schematic illustration of culture techniques for renal cell**s**. (a) When tubule segments are cultured in medium containing serum, cells emigrate and spread on the bottom of the culture dish and on the outer side of the tubule basal lamina. (b) To offer a substitute for the basal lamina, renal cells are kept on various filter materials supporting exchange of fluid between the luminal and basal side**s**. (c) A fully biocompatible surface on hollow fibers is needed, when renal cells have to perform intense transport of molecules. (d) Culture of an intact organ anlage promotes spatial development of tubules. A glass capillary for perfusion of medium supports nutrition during long-term culturing. (e) Development of structured tubules is obtained in transfilter experiments with isolated nephrogenic mesenchyme. (f) Coating of single cells by extracellular matrix proteins promotes the spatial formation of tubules. However, during long-term culturing the coating of extracellular matrix hinders the exchange of nutrients, including respiratory gas.

For example, segments of renal tubules were isolated and placed at the bottom of a culture dish [21,22]. Most interestingly, application of a culture medium containing fetal bovine serum does not result in the elongation of the isolated tubule (Figure 1a). Instead, cells leave the interior of the tubule and proliferate on the bottom of the culture dish and on the outer surface of the tubular basal lamina. It is unknown why the impermeable polystyrene of the dish is more attractive for renal cells than to stay in the interior of the tubule.

To investigate mechanisms of adhesion, renal cells were cultured on various biomaterials. In all of the cases, it was observed that renal cells react with unexpected sensitivity when they come into contact with a biomaterial. Thereby, multiple biophysical, biochemical and cell biological properties determine if the complete surface of the selected biomaterial is accepted for adhesion or if only part of it is covered by proliferating cells [23,24,25].

Not only proliferation and adhesion of renal cells is of importance but also their degree of differentiation after several days of culturing. To offer a substitute for the basal lamina, renal cells were kept on various filter materials supporting exchange of fluid and molecules between the luminal and basal sides (Figure 1b) [26,27,28]. These experiments also revealed that the material and the pore size of the selected filter has an enormous influence on the degree of cell proliferation, adhesion, polarization and subsequent differentiation. Futhermore, it was demonstrated that only a fully biocompatible surface as a basal lamina substitute supports functional differentiation of renal epithelial cells. This is of special importance when renal cells are cultured on hollow fibers to fulfill an intense transepithelial transport of molecules (Figure 1c) [29].

### 1.3. From cells to parenchyma

A monolayer of cells on the bottom of a culture dish or on a filter membrane is a nice model for testing viability, influence of drugs and transport routes. However, it does not reflect the situation when regeneration of parenchyma is being investigated. To obtain insights in the development of parenchyma, intact metanephric organ anlagen were isolated and cultured on a filter (Figure 1d) [30,31]. To facilitate exchange of respiratory gas, the tissue was kept near the gas-fluid interface with a minimal overlay of culture medium. At the start of the culture, the integrity of the organ anlage promotes spatial development of tubules. However, the continuously increasing layers of parenchyma finally hinder provision with fresh nutrition and respiratory gas, which in turn limits further extension of the organ. For that reason, a glass capillary for perfusion of medium was placed inside the hilus to prolong the period of growth during culture [12].

To obtain primary information about the developmental potential of renal stem/progenitor cells, nephrogenic mesenchyme was isolated, placed on a nitrocellulose filter and coated by agarose (Figure 1e) [32,33]. Then the filter was turned to mount living spinal cord from the same fetus as an embryonic inducer. The contact between both tissues through the filter pores results in the development of structured tubules within the mesenchymal layer. By performing these kind of experiments, it was recognized that numerous reciprocal molecular interactions occur during renal organogenesis resulting finally in nephron formation including tubules with specific differentiation [34,35].

In addition to the use of intact tissue, isolated cells were used to investigate cell biological mechanisms involved in tubule formation. For example, cells collected from the urine [36] and MDCK cells [37] were coated before culture by extracellular matrix proteins such as collagen [38], matrigel^®^ [39], hydrogel [37] and hyaluronic acid [40]. In all of these experiments, it was demonstrated that coating by extracellular matrix proteins promotes the spatial formation of tubules (Figure 1f). However, it was also observed that during long-term culturing, the coat of extracellular matrix leads to the formation of unstirred layers of fluid hindering the exchange of nutrients including respiratory gas. In turn, this situation causes deleterious accumulation of metabolites, thus limiting the period of culture time.

## 2. View to the Embryonic Parenchyma

### 2.1. The renal stem cell niche

**Figure 2:**
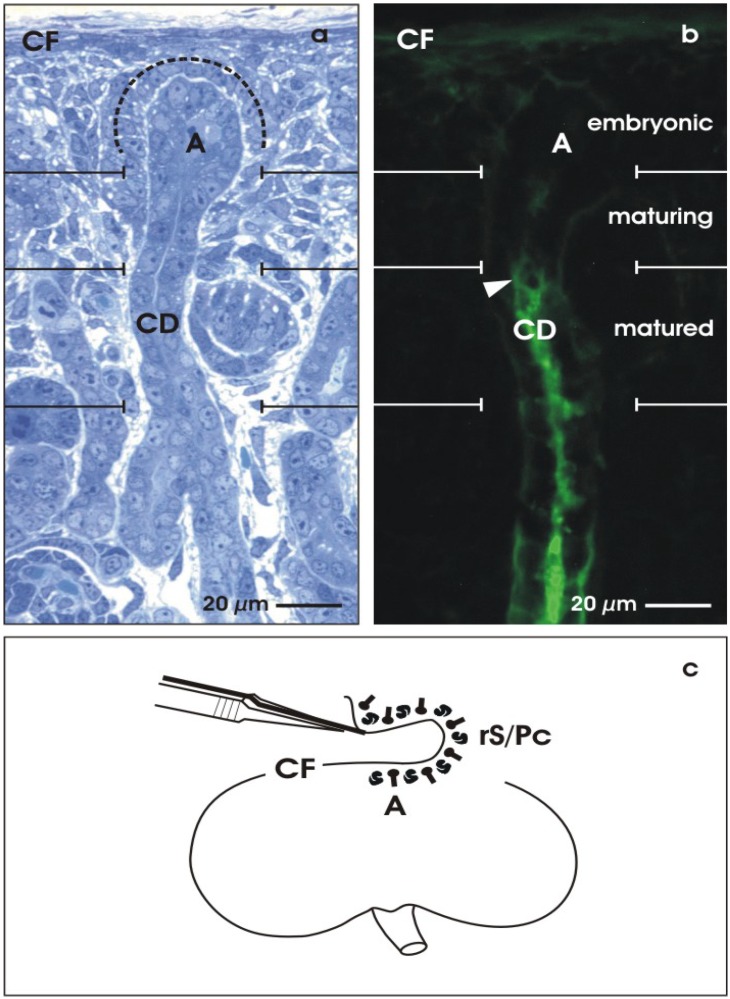
View to the niche of renal stem/progenitor cells. The outer cortex of neonatal rabbit kidney can be divided into an embryonic, maturing and matured zone. (a) Semi-thin section and (b) cryosection. (a) Underneath the capsula fibrosa (CF), epithelial stem/progenitor cells are found within the ampulla (A) tip of the collecting duct (CD). Nephrogenic mesenchymal stem/progenitor cells (dotted line) surround the basal aspect of the ampulla. (b) Soybean Agglutinin (SBA)-label demonstrates that the epithelial stem/progenitor cells within the ampulla are not recognized by lectin, while the maturing neck shows the first signs of cellular reaction. Intensive label is found in the matured collecting duct (arrow head). (c) Isolation of renal stem/progenitor cells by stripping off the renal CF using fine forceps. The adherent layer of embryonic tissue contains collecting duct ampullae (A) and nephrogenic mesenchymal stem/progenitor cells (rS/Pc).

Regeneration appears as a recovery of parenchyma, including embryonic and adult developmental processes. For a better understanding, sound knowledge about nephrogenesis is required. The induction of renal tubules occurs within a unique renal stem/progenitor cell (rS/Pc) niche. This special site is easily accessible in the kidney of newborn rabbits, where nephrogenesis takes place still in the neonatal phase. During ongoing organ development, the niche of stem/progenitor cells is permanently located underneath the organ capsule (Figure 2a) [41,42]. Step-by-step, new generations of nephrons are induced here. In parallel to this process, the renal parenchyma extends in a radial direction and in close vicinity to the organ capsule. Thus, underneath the organ capsule, nephrogenesis has started in the stage of organ anlage. After the organ has reached its final size, nephrogenesis is terminated here by an unknown molecular mechanism in which degradation of renal stem/progenitor cells, ureteric bud cell lineages or a change of the gene expression in response to a physiological sensor could be involved [43].

Two different kinds of cells are localized within the renal stem/progenitor cell niche [44,45]. The tip of the collecting duct (CD) ampulla (A) contains epithelial stem/progenitor cells originally derived from the ureter bud. Mesenchymal nephrogenic stem/progenitor cells (dotted line) surround the basal aspect of each collecting duct ampulla. Reciprocal interactions between both cell populations leads to a condensation of nephrogenic stem/progenitor cells, resulting in a Comma-shaped and then in a S-shaped body as first visible signs of nephron development. The capsule (CF)-orientated wing of a S-shaped body develops into proximal, intermediate and distal tubule segments of the nephron, while the medulla-orientated wing forms the glomerulus.

Except for PI 3-kinase and mTOR signaling pathways, the molecular mechanisms leading to different tubule segments are unknown [46]. Also, the preceding formation from the S-shaped body into spatially organized tubules is currently not understood. This step comprises the sprouting of cells to reach the necessary amount, the formation of a lumen and the extension of the individual segment. In a parallel process, a polarized epithelium is integrated into the arising tubule exhibiting exact geometrical dimensions such as course, length, inner and outer diameter [47,48,49,50].

### 2.2. Maturation of stem/progenitor cells within the collecting duct ampulla

Due to the limited size of embryonic mouse or rat specimens, neonatal rabbit kidney is selected as a favorite resource for stem/progenitor cells (rS/Pc) in the presented investigation [51]. Even after birth, the cortex of the growing kidney contains numerous stem cell niches within the original extracellular matrix environment. To illustrate proceding development, a vertical cryosection through the cortex of a neonatal rabbit kidney was labeled by Soybean Agglutinin (SBA) (Figure 2b). Progress of development can be recognized by comparing the embryonic, maturing and matured zones of the outer cortex. For example, within the collecting duct ampulla (A), embryonic cells are lacking SBA-label, while in the neck (maturing) some label is observed and in the shaft (matured, arrow head) an intensive cellular reaction is found. In addition, the cortico-medullary course of the developmental gradient within the kidney can be used to compare the attainable degree of differentiation found for example in generated collecting duct (CD) tubules.

### 2.3. Isolation of renal stem/progenitor cells

For the presented experiments, rS/Pc were isolated to investigate their potential for spatial tubule development under advanced culture conditions. The cortex of neonatal rabbit kidney appears as an ideal source for this kind of experiments. Stripping off the CF with fine forceps, a thin layer of embryonic tissue adheres to the explant (Figure 2c) [51]. The isolated tissue layer contains numerous epithelial rS/Pc included in the tip of the collecting duct ampulla and adherent nephrogenic mesenchymal rS/Pc. By this simple isolation method, an embryonic tissue layer of up to one square cm can be harvested. Up until now, no other species is known for the isolation of renal stem/progenitor cells in such an amount.

## 3. The New Concept—Offering an Artificial Interstitium

### 3.1. Environment for spatial development

The aim of the presented experiments was to find a culture ambience that meets the physiological needs of rS/Pc so that they can be stimulated to form tubules. To obtain exact information about the involved developmental processes, culture has to be performed under exactly defined conditions [52]. For that reason, addition of fetal bovine serum to the medium, use of unspecified morphogenic factors and coating by extracellular matrix proteins is omitted. Moreover, the culture system should deliver the necessary amount of tissue required for subsequent cell biological analysis.

**Figure 3:**
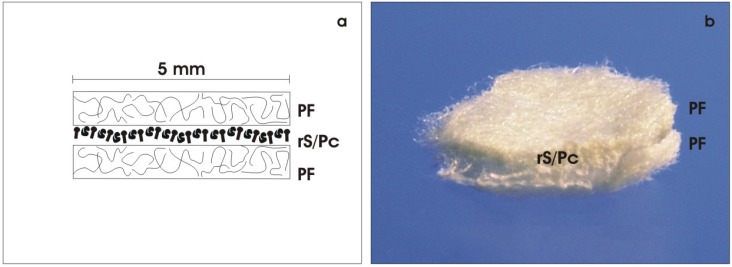
Creation of an artificial interstitium. (a) An artificial interstitium for the culture of renal stem/progenitor cells (rS/Pc) is made by mounting the isolated embryonic tissue between two layers of polyester fleece (PF). (b) Photographic illustration of a basic sandwich set-up containing isolated embryonic tissue with rS/Pc between layers of PF.

Thus, to fulfil all these demands, an innovative technique was elaborated [53,54,55]. In analogy to the kidney, an artificial interstitium was created supporting the organization of rS/Pc so that formation of tubules can occur [56]. The technical solution is to mount renal stem/progenitor cells between layers of a polyester fleece (Figure 3). This technique promotes spatial development and compensates coating by extracellular matrix proteins. The fluid network between polyester fibers prevents formation of unstirred layers of culture medium, so that continuous provision of nutrients and respiratory gas becomes possible.

In practice, an artificial interstitium is created by placing the isolated embryonic renal tissue between two punched out layers of polyester fleece (I7, Walraf, Grevenbroich, Germany) measuring 5 mm in diameter and up to 250 µm in height (Figure 3a). This arrangement results in a basic sandwich set-up configuration with the freshly isolated embryonic tissue in the middle and layers of polyester fleece covering the outer sides (Figure 3b). As illustrated later, the interface between the layers of polyester fleece exhibit biophysical features that promote the spatial formation of tubules.

### 3.2. Protecting the developing tissue

**Figure 4:**
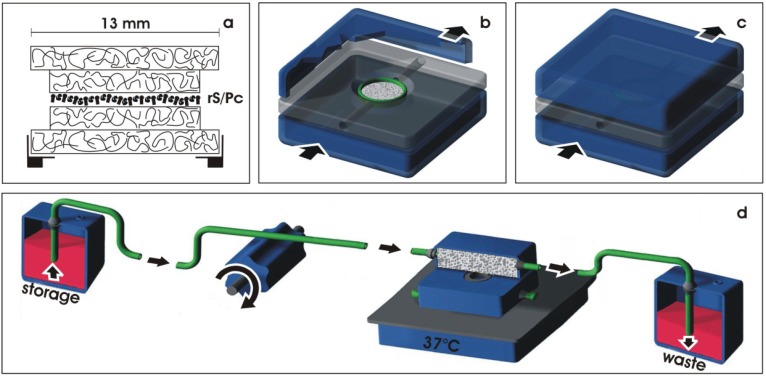
Set-up for perfusion culture. (a) Basic sandwich set-up containing renal stem/progenitor cells (rS/Pc) is mounted in a tissue carrier. (b) For culturing, the tissue carrier is placed at the base of a perfusion container. (c) After closing the lid, the tissue carrier is held in position. (d) Perfusion culturing is performed with a peristaltic pump transporting (arrow) always fresh medium (1.25 mL/h) from the storage bottle to the culture container. Used medium leaving the culture container is not recycled but is collected in a waste bottle.

To prevent damage to developing tubules during long-term culturing, the basic sandwich set-up of 5 mm in diameter containing rS/Pc is mounted in a tissue carrier (Figure 4a). First, a layer of polyester fleece measuring 13 mm in diameter is placed in a base ring of a Minusheet® tissue carrier (Minucells and Minutissue, Bad Abbach, Germany). Then the sandwich set-up containing rS/Pc is mounted and covered by an additional layer of polyester fleece measuring 13 mm in diameter. For long-term culturing, the tissue carrier is transferred to a perfusion culture container with horizontal flow characteristics (Figure 4b) (Minucells and Minutissue). After closing the lid of the container, the basic sandwich set-up is held in an exact position (Figure 4c). For culturing, the container is connected over silicone tubes with a storage and waste bottle (Figure 4d). To maintain a constant temperature of 37 °C, the perfusion culture container is placed on a thermoplate (Medax-Nagel, Kiel, Germany) and covered with a transparent lid (not shown).

### 3.3. Transport of culture medium

During the experimental run of 13 days, constant transport of medium is performed (Figure 4d). Always fresh and chemically defined IMDM (Iscove´s Modified Dulbecco´s Medium including Phenolred, GIBCO/Invitrogen, Karlsruhe, Germany) is transported at a rate of 1.25 mL/h with an IPC N8 peristaltic pump (Ismatec, Wertheim, Germany).

Applying this kind of perfusion technique medium is saturated to 160 mmHg oxygen during transport. This high content of oxygen in the medium is reached by a long and thin-walled silicone tube, which is highly gas-permeable and ensures optimal diffusion between culture medium and surrounding atmosphere. It guarantees an optimal supply of respiratory gas for the growing tissue. In this way it is possible to adjust the gas partial pressures within the medium under absolutely sterile conditions. In order to maintain a constant pH of 7.4 under atmospheric air containing 0.3% CO2, HEPES (50 mmol/l, GIBCO) is added to the medium.

To induce tubulogenic development in rS/Pc during the whole culture period, aldosterone (1 × 10^-7^ M, Fluka, Taufkirchen, Germany) is administered to the medium. Infections are prevented by adding an antibiotic-antimycotic cocktail (1%, GIBCO).

## 4. Experiments on Generated Tubules

### 4.1. Labeling whole mount specimens

**Figure 5:**
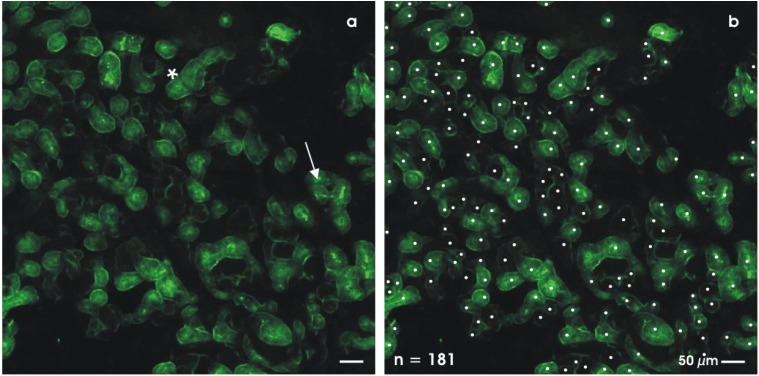
Generated renal tubules at the interface of an artificial polyester interstitium after 13 days of perfusion culture. (a) Fluorescence microscopy of whole mount specimens labeled by SBA. Tubules exhibit a lumen (arrow) and a basal lamina (asterisk). (b) Label reveals that in this specimen 181 tubules can be detected.

After a culture period of 13 days, the artificial interstitium can be opened by tearing off the layers of polyester fleece. To analyze the spatial extension of tubules, specimens were fixed in 70% ethanol, labeled by SBA and analyzed by fluorescence microscopy (Figure 5a). At the start of culturing, isolated rS/Pc do not exhibit cellular SBA-label (Figure 2b, A). In contrast, after a culture period of 13 days, intense binding of SBA is recognized on numerous generated tubules (Figure 5a). The up-regulation of SBA label signals a step in differentiation, as it is described for the neonatal kidney (Figure 2b).

The surface view depicts that numerous tubules are growing in a spatial manner (Figure 5a). Part of the generated tubules exhibit a straight growth, while others reveal a dichotomous branching or curling. All of generated tubules show polarized cells, a visible lumen and a basal lamina. The number of tubules can be determined using a WCIF ImageJ program (Bethesda, Maryland, USA) by counting each SBA-labeled tubule (Figure 5b). Applying this technique, an individual specimen reveals 181 (white spots) tubules that can be detected within a microscopic opening of 790 × 790 μm. When the tubules do not leave the optical plain, it is possible to follow their longitudinal growth over a distance between 300 and 400 μm.

### 4.2. Detecting cell biological differentiation

**Figure 6:**
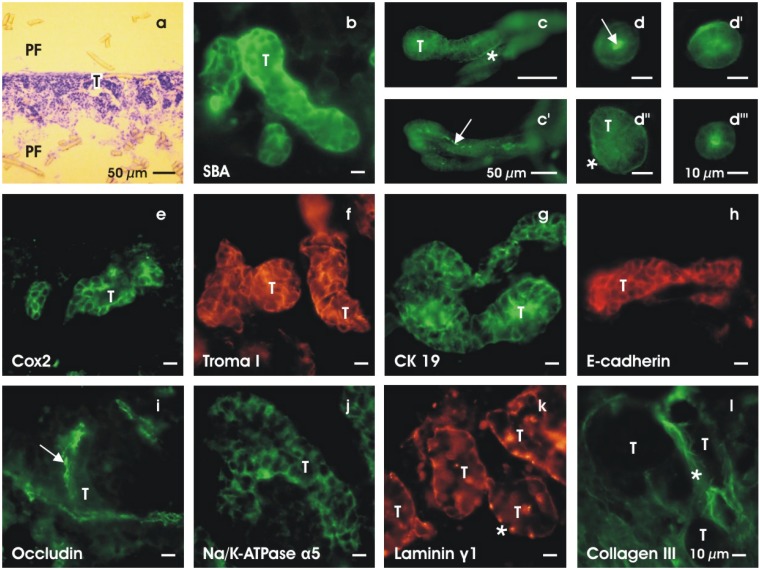
Cell biological features of tubules generated for 13 days at the interface of an artificial interstitium. (a) Toluidin blue stain of a cryosection demonstrates numerous tubules (T) growing between polyester fleeces (PF) at the upper and lower side. (b) SBA-label depicts a tubule (T) in spatial extension. **(c,c’and d,d’,d’’,d’’’)** Label for SBA shows that tubules (T) exhibit a lumen (arrow) and a basal lamina (asterisk). Immuno-label for (e) cyclooxygenase 2 (Cox2), (f) cytokeratin endo A (Troma I), (g) cytokeratin 19 (CK19), (h) E-cadherin, (i) occludin (arrow), (j) Na/K-ATPase α5 and (k) laminin γ1 reveals intensive reaction on cultured specimens. (l) Label for collagen III (asterisk) is found at the basal lamina and in the interstitial space between tubules (T).

Besides the spatial distribution, the degree of acquired cell biological differentiation within generated tubules is of fundamental importance. To analyze features of differentiation, immunohistochemistry on cryosections was performed (Figure 6b-l). For control, the surface view of a Toluidin blue stained section demonstrates the distribution of developed tubules (T) covered by layers of polyester fleece (PF) on the upper and lower sides (Figure 6a). View to SBA-labeled specimens in higher magnification shows tubules (T) in a spatial arrangement (Figure 6b). A longitudinal view to SBA-labeled tubules exhibits a distinct lumen (arrow) and a basal lamina (asterisk) (Figure 6c, c’). The vertical view to SBA-labeled tubules depicts in the center a lumen, while at the basal aspect a basal lamina can be seen (Figure 6d,d’,d’’,d’’’).

Immunohistochemistry for cyclooxygenase 2 (Cox2, Figure 6e), cytokeratin endo A (Troma I, Figure 6f), cytokeratin 19 (CK 19, Figure 6g) and E-cadherin (Figure 6h) exhibits intensive label within all of the tubule cells. Label for occludin (arrow) demonstrates the development of a tight junctional belt recognized as faint label in the luminal portion of generated tubules (Figure 6i). Immuno-label for Na/K-ATPase α5 reveals an intensive fluorescence at the basolateral aspect (Figure 6j). Labeling the tubules (T) for laminin γ1 exhibits an intensive reaction at the basal lamina (asterisk) (Figure 6k). All of these immunohistochemical data speak in favor that the generated tubules contain a polarized epithelium with functional features resembling the adult renal collecting duct tubule.

The immuno-label on cryosections further reveals that collagen type III is contained in both the basal lamina (asterisk) of generated tubules (T) and in the surrounding interstitial space standing in contact with polyester fibers of the artificial interstitium (Figure 6l). This result speaks in favour that the differentiation of tubules appears strongly correlated with the synthesis of an intact basal lamina containing laminin γ1 and a typical interstitial protein such as collagen type III.

### 4.3. Regarding ultrastructural features

The presented tubules were generated without coating them by extracellular matrix proteins. For that reason, it is possible to analyze the basal aspect of developing tubules by scanning electron microscopy (SEM) without the interference of proteins derived from a coating process [57,58].

**Figure 7:**
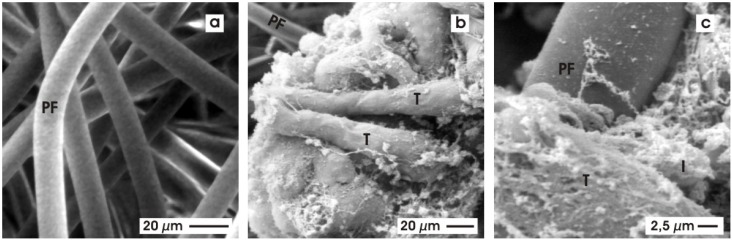
View by scanning electron microscopy (SEM). (a) The fleece shows numerous polyester fibers (PF) running in a longitudinal, transversal and oblique course. (b) Illustration depicts growing tubules (T) in the vicinity of PF. **(c)** On the surface of tubules (T), single interstitial cells (I) and thin fibers consisting of extracellular matrix are observed.

SEM analysis of the used polyester fleece (PF) shows that the numerous fibers are running in a longitudinal, transversal and oblique course (Figure 7a). They appear to be of homogeneous composition and exhibit a smooth surface without recognizable protrusions or roughness. The average diameter of a polyester fiber is 10 μm. Chemical crosslinking between them cannot be observed.

The area of the polyester fleece used for tissue development exhibits numerous tubules (T, Figure 7b). Part of it grows in a parallel fashion in the vicinity of the polyester fleece fibers (PF), some of them show a curling course, while others exhibit a dichotomous branching. All of the tubules are covered by a continuously developed basal lamina. Higher magnification further demonstrates that the tubules have an apparently light contact to the fibers of the polyester fleece (Figure 7c). On the surface of the tubules, single interstitial cells (I) and bundles of newly synthesized extracellular matrix proteins are recognized.

**Figure 8:**
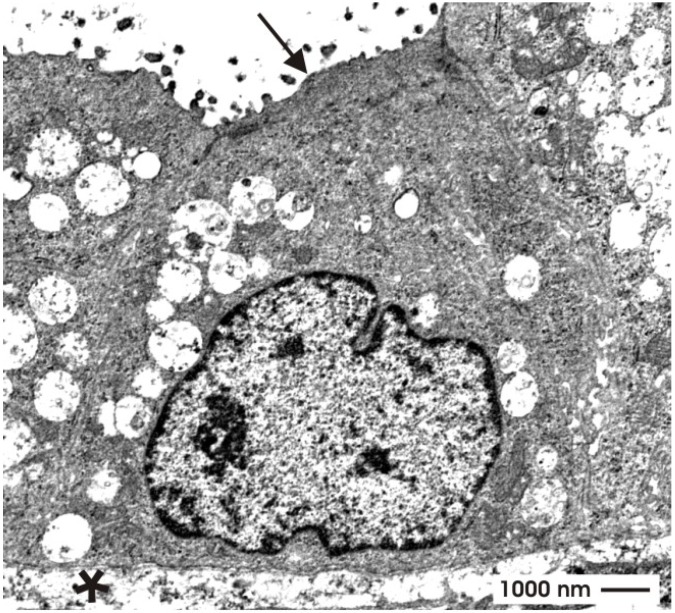
View by transmission electron microscopy (TEM). Illustration demonstrates that generated tubules contain a polarized epithelium. The apical side borders a lumen (arrow), while the basal side contains a basal lamina (asterisk). Between the apical and lateral plasma membrane a tight junctional complex is developed.

To obtain further insights into the quality of differentiation in tubule cells transmission electron microscopy (TEM) was performed [59]. Low magnification reveals that generated tubules contain a lining epithelium with a clearly visible lumen (arrow) and a constantly developed basal lamina (asterisk). In the surrounding of tubules synthesized extracellular matrix, single interstitial cells and some debris is noticed. Higher magnification reveals that an isoprismatic epithelium is established within generated tubules (Figure 8). Most important, the luminal and lateral plasma membranes are separated by a typical tight junctional complex. It consists of a zonula occludens, zonula adhaerens and a desmosome. The cells exhibit a large nucleus, which is located in the center of the cytoplasm. In the apical and basal cytoplasm numerous lysosomal elements are found. Small, medium-sized and large vacuoles are filled to a various degree with electron-dense material. The vacuoles suggest that the containing material has been phagocytosed. At the basal aspect of the epithelium a basal lamina is found consisting of a lamina rara interna, a lamina densa and an extended lamina fibroreticularis. Summing up, all of the ultrastructural data (Figure 7 and Figure 8), but also the immunohistochemical results (Figure 6i,j) point out that during culture a polarized and an obviously sealing epithelium is developing within generated tubules.

## 5. Triggering Formation of Tubules

### 5.1. Inducing tubulogenic development

**Figure 9:**
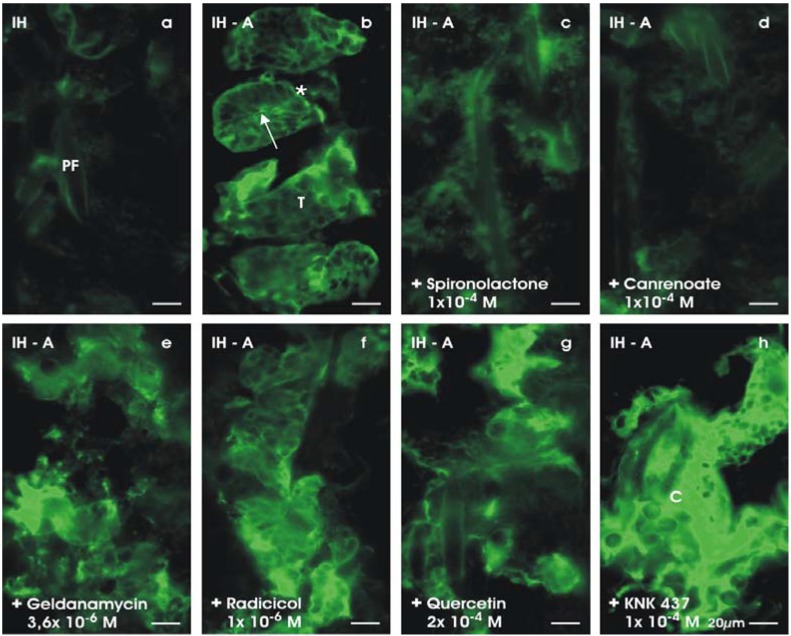
Interfering the tubulogenic effect of aldosterone. (a) Generation of SBA-labeled tubules cannot be recognized if aldosterone is omitted. (b) In contrast, numerous tubules (T) are observed after application of aldosterone (1 × 10^-7^ M). Arrow shows lumen, while asterisk depicts basal lamina. (c) Application of 1 × 10^-4^ M spironolactone or (d) 1 × 10^-4^ M canrenoate in the presence of aldosterone completely inhibits the development of tubules. (e) Aldosterone in combination with geldanamycin (3.6 × 10^-6^ M) or (f) radicicol (1 × 10^-6^ M) does not result in the formation of tubules, but leads to multiple SBA-labeled cell clusters. (g) Administration of aldosterone in the presence of quercetin (2 × 10^-4^ M) or (h) KNK 437 (1 × 10^-4^ M) inhibits development of tubules but shows intensively labeled cell clusters (C).

When the culture of renal stem/progenitor cells is performed in medium lacking aldosterone, SBA-labeled tubules are not developed. Instead a disintegration of the isolated embryonic tissue is noticed (Figure 9a). In contrast, when aldosterone (1 × 10^-7^ M) is administered to the standard medium (IMDM), a complete change in the developmental pattern of renal stem/progenitor cells occurs (Figure 9b). The steroid hormone induces the formation of numerous SBA-labeled tubules within a culture period of 13 days [53,57,60,61].

To obtain better insights in the tubulogenic effect of aldosterone, the hormone was applied in concentrations ranging from 1 × 10^-10^ M to 1 × 10^-5^ M [53]. A first series of experiments revealed that a low dose of aldosterone (1 × 10^-10^ M) does not stimulate the development of tubules, while concentrations of 1 × 10^-9^ M and 1 × 10^-8^ M start to induce outgrowth of SBA-labeled cells that form long rows and clusters but not structured tubules. Intact formation of tubules is obtained by the use of 1 × 10^-7^ M and 1 × 10^-6^ M aldosterone. Surprisingly, an application of a higher dose such as 1 × 10^-5^ M does not better stimulate the development of tubules.

### 5.2. Specifity of aldosterone action

The synthesis of aldosterone starts from cholesterol, which is metabolized over pregnenolone to progesterone, 11-deoxycorticosterone, corticosterone and 18-hydroxycorticosterone. From the adult kidney it is known that not only aldosterone, but also some of its molecular precursors, reveal a high affinity to the mineralocorticoid receptor (MR), influencing thereby physiological functions [61]. Experiments on adult kidney showed, for example, that 11-deoxycorticosterone is as effective as aldosterone, while corticosterone is 100-times less potent.

In the next series of experiments, it was investigated if also precursors (each 1 × 10^-7^ M) of the aldosterone synthesis pathway exhibit a tubulogenic effect on rS/Pc .

When testing precursors of aldosterone synthesis, it was recognized that application of cholesterol or pregnenolone does not induce the formation of any SBA-positive tubule. Treatment with progesterone leads to the development of single tubules with low SBA-label. When 11-deoxycorticosterone is used, only few tubules with a faint SBA-label can be detected. In contrast, administration of corticosterone does not exhibit any development of tubules. Instead, numerous SBA-labeled cell clusters are observed in close contact to polyester fibers. Data for 18-hydroxycorticosterone are missing, since this substance was not commercially available. Most impressive, only the administration of aldosterone is leading to numerous SBA-labeled tubules exhibiting a distinct lumen and a clearly recognizable basal lamina [57].

The tubulogenic action of aldosterone might be triggered in cooperation *via* the glucocorticoid receptor (GR). Consequently, the glucocorticoid dexamethasone instead of aldosterone was applied. This series of experiments shows that administration of aldosterone (1 × 10^-7^ M) results in the development of numerous SBA-labeled tubules, while the use of dexamethasone (1 × 10^-7^ M) produces widely distributed clusters of non-polarized cells. In consequence, the process of tubule development is highly dependent on the administration of aldosterone (1 × 10^-7^ M).

### 5.3. Antagonizing the action of aldosterone

The previously performed experiments demonstrate that aldosterone is required to induce a tubulogenic development in isolated renal stem/progenitor cells. However, the data do not reveal, if the tubulogenic effect of aldosterone is mediated *via* the mineralocorticoid receptor (MR) or if it is related to an unspecific side effect. To obtain more information about the specific binding of aldosterone, the influence of antagonists such as spironolactone and canrenoate was tested.

For comparison, omittance of aldosterone does not show generation of tubules (Figure 9a), while application of aldosterone (1 × 10^-7^ M) is resulting in numerous tubules (Figure 9b). Application of a low dose of spironolactone (1 × 10^-7^ M) in the presence of aldosterone (1 × 10^-7^ M) does not affect the development of SBA-labeled tubules. However, use of a higher concentration of spironolactone (1 × 10^-5^ M) demonstrates inhibitory effects that lead to a switch in development. The number of structured tubules is reduced and SBA-labeled cells start to form extended cell clusters. Application of 1 × 10^-4^ M spironolactone completely prevents the development of SBA-labeled tubules (Figure 9c) [60].

In a further series of experiments, the antagonist canrenoate was tested (Figure 9d). The results show a same concentration-dependent effect on the tubulogenic action of aldosterone, as was observed with spironolactone. Application of 1 × 10^-7^ M canrenoate in the aldosterone-containing medium does not affect the development of tubules, while administration of 1 × 10^-6^ M and 1 × 10^-5^ M canrenoate drastically reduces SBA-labeled cells. Finally, the use of 1 × 10^-4^ M canrenoate results in a complete lack of SBA-labeled cells and tubules (Figure 9d). Thus, the simultaneous administration of aldosterone in combination with spironolactone or canrenoate demonstrates that the tubulogenic effect is inhibited in a dose dependent manner. This result also clearly depicts that the tubulogenic effect of aldosterone is not an unspecific reaction or a side effect on rS/Pc, but is mediated specifically *via* the mineralocorticoid receptor (MR) [61].

### 5.4. Mineralocorticoid receptor and chaperons

In a next set of experiments, intracellular processing of the tubulogenic effect of aldosterone was investigated. On the one hand MR may be randomly distributed within the cytoplasm of the target cells, on the other hand information is available that MR is standing in close molecular contact with heat shock proteins (hsp) 90 and 70 [62]. In so far, the tubulogenic effect of aldosterone can be abolished when rS/Pc are treated with substances that disrupt these interactions.

To interfere with contact between MR and hsp 90, rS/Pc were cultured in IMDM containing geldanamycin (3.6 × 10^-6^ M) in combination with aldosterone (1 × 10^-7^ M) for 13 days (Figure 9e). Geldanamycin specifically binds to hsp 90 thereby blocking the ATP-binding site due to its higher affinity compared to ATP. In this way, geldanamycin hinders the contact between hsp 90 and activated MR [63]. The performed culture experiments reveal that structured tubules cannot be found after geldanamycin treatment. Instead, numerous SBA-labeled cells are localized within extended clusters.

Furthermore, radicicol is a macrocyclic antifungal substance that binds in the same way as geldanamycin [64]. It hinders ATP-dependent conformational changes that are required for cytoplasmic interactions between target proteins such as MR. Culturing of rS/Pc with radicicol (1 × 10^-6^ M) in combination with aldosterone (1 × 10^-7^ M) produces only few structured tubules, but numerous SBA-labeled cells in form of extended clusters (Figure 9f). In consequence, experiments with both geldanamycin and radicicol show that the tubulogenic effect of aldosterone is missing, when the molecular contact between MR and hsp 90 is disturbed [65].

Interfering the tubulogenic signal at the level of hsp 70 by quercetin (2 × 10^-4^ M) in combination with aldosterone (1 × 10^-7^ M) results in numerous SBA-labeled cells contained within extended clusters, while only minimal development of tubules can be detected (Figure 9g) [66]. KNK 437 is a benzylidene lactam molecule [67] that inhibits heat shock factor activity, resulting in a decreased expression of heat shock proteins, thereby interfering indirectly with MR. Finally, performing culture experiments with KNK 437 (1 × 10^-4^ M) in combination with aldosterone (1 × 10^-7^ M) demonstrate that development of tubules is lacking, but numerous SBA-labeled cells are found within extended clusters (Figure 9h).

Thus, all of the presented data point out that aldosterone stimulates the formation of tubules when an intact contact between MR and related chaperons hsp 90 and 70 is given [65]. In contrast, when the contact between MR and chaperons is disrupted, the tubulogenic effect of aldosterone turns into the formation of extended SBA-labeled cell clusters. In consequence, the development of cell clusters instead of tubules reflects a malformation. Such a development has to be prevented when rS/Pc are applied as an implant to promote a process of regeneration in a diseased kidney.

## 6. Considerations about Implantation

### 6.1. Piling of renal stem/progenitor cells

Present experiments reveal that culture of renal stem/progenitor cells at the interface of an artificial interstitium appears as an ideal model to investigate stimulating and inhibiting influences of morphogenic drugs and innovative biomaterials on the spatial development of renal tubules. In addition, a controllable growth of tubules at an artificial interstitium might be the base for a successful implantation of stem/progenitor cells into the kidney to regenerate diseased parenchyma.

However, the therapeutic realization is a complex task and needs a multidisciplinary approach. Following this strategy, much more basic research in the area of spatial tubule formation must be performed. For a therapeutic application, one has to find out the right source of human stem/progenitor cells, one has to elaborate a standardized procedure for mounting the best concentration of cells on a biodegradable fleece material, one has to learn about the optimal site of implantation and finally one has to find a method to promote rapid microvascularization in parallel to the starting regeneration of parenchyma.

A special issue in this context is to implant the correct amount of stem/progenitor cells [68]. A shortage may lead to minor or lacking regeneration, while abundance may provoke cluster formation as a result of malformation. Furthermore, it has to be elaborated, if stem/progenitor cells can be injected in form of accidental spots or if they must be implanted at a special site, in a spatial order and in a certain density.

**Figure 10:**
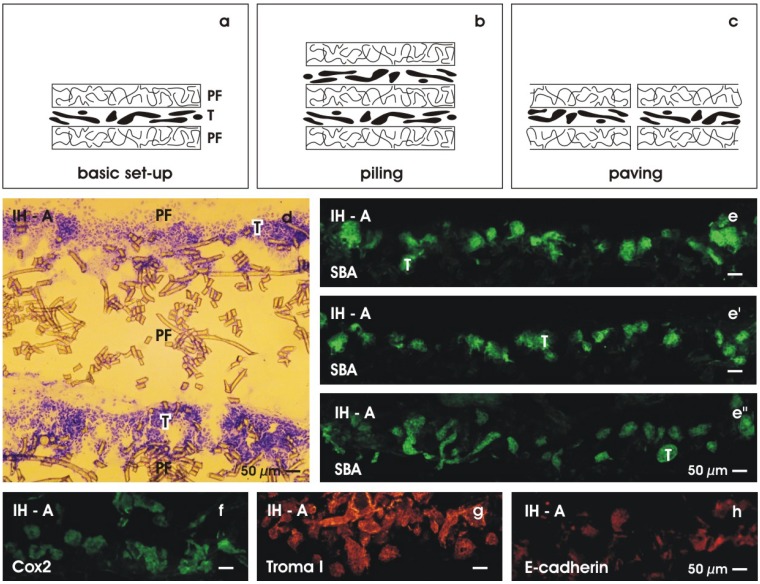
Guiding the spatial extension of generated tubules. (a) An artificial interstitium is created by mounting generated tubules (T) between layers of polyester fleece (PF). (b) Layers of tissue can be increased by piling. (c) The basic sandwich set-up configuration can also be used to extend the area of growth by paving. (d) Toluidin blue stained cryosection demonstrates two rows of tubules (T) between polyester fibers (PF) in a piled specimens. (e, e’, e’’) SBA-label of tubules generated in piled specimens. Immuno-label for (f) cyclooxygenase 2 (Cox2), (g) Troma I, (h) E-cadherin.

Implantation of stem/progenitor cells in a spatial orientation could be solved by mounting them between layers of fleece (Figure 10a). The specific sandwich set-up configuration makes it possible to adapt individually the necessary amount of rS/Pc by piling (Figure 10b) and/or paving (Figure 10c) [69]. To demonstrate the feasibility of extending systematically the spatial extension of tubules, in a last set of experiments, rS/Pc were piled between layers of polyester fleece at the begin of perfusion culturing (Figure 10b). The spatial extension of tubules was investigated 13 days later. A cryosection labeled by Toluidin blue shows development of two parallel rows of tubules, each of them separated at the top and base by layers of polyester fleece (Figure 10d). Labeling generated tubules by SBA (Figure 10e, e’, e’’), cyclooxygenase 2 (Cox2) (Figure 10f), Troma I (cytokeratin endo A, Figure 10g) and E-cadherin (Figure 10h) reveals that the same amount and an identical degree of differentiation is found in piled specimens as observed in experiments with a single row of generated tubules. In so far presented experiments exhibit that piling of basic sandwich set-ups like bricks is principally feasible and might be a fruitful perspective for guiding the spatial growth of tubules, when a subcapsular implantation into the kidney is under consideration.

### 6.2. Finding a solution for rapid micro-vascularization

**Figure 11:**
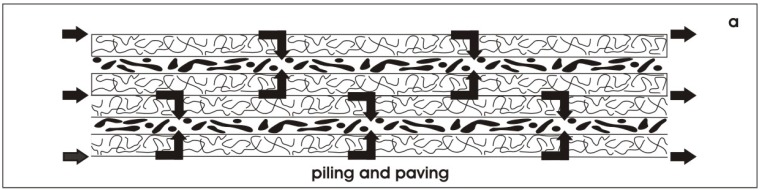
The space between the polyester fibers in piled and paved specimens could be used for supply of culture medium. Arrows indicate the possible flow of medium and respiratory gas during perfusion culture.

When piling and paving is performed, rS/Pc are mounted between layers of polyester fleece. Thus, each row of rS/Pc is separated by the fibers of the fleece. However, despite this spatial separation, all of the tissue rows are connected by a network of fluid. Offering an artificial polyester interstitium culture medium is transported in the space between the fleece fibers guaranteeing at each site constant nutrition and respiratory gas (Figure 11). Moreover, the space between the fibers can be used to support microvascularization. For example, endothelial cells could be added to the culture medium and transported along the fluid network. In the case of a subcapsular implantation a widely distributed net of endothelial cells is available that may promote vascularization in shortest time and in parallel to a starting regeneration of tubules.

### 6.3. Using basic sandwich set-ups as a subcapsular implant

**Figure 12:**
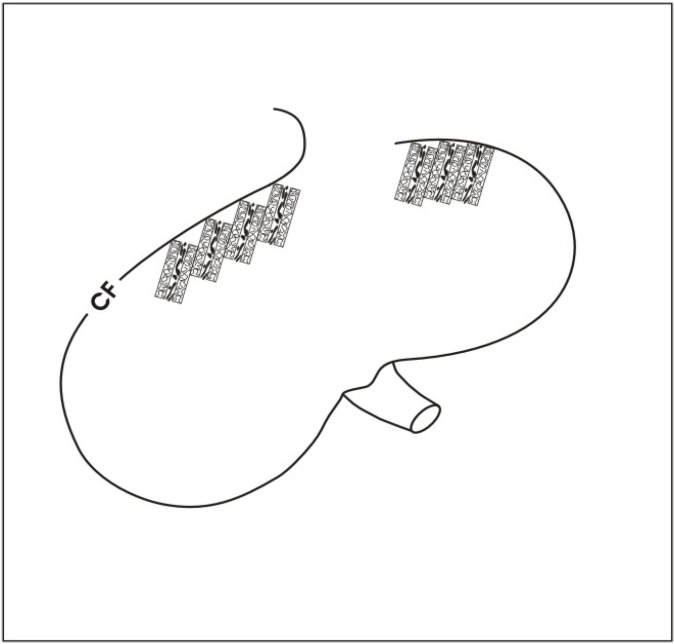
Schematic illustration of possible implantation site. Basic sandwich set-ups can be stacked underneath the renal capsule. At this specific site, nephrogenesis has been terminated once the growing kidney has reached its final size. The idea is to re-activate the site of nephrogenesis by the implantation of renal stem/progenitor cells.

A challenge for a future biomedical application is to find the optimal site for an implantation of human stem/progenitor cells. Applying basic sandwich set-ups we favour an implantation underneath the renal capsule as it was earlier successfully performed with other tissues [70,71]. The cortex corticis of parenchyma underneath the renal capsule is the site, where nephrogenesis has been terminated at the end of organ growth. In consequence, the idea is to re-activate the earlier site of nephrogenesis by the implantation of stem/progenitor cells. Furthermore, the pouch underneath the capsule is easily accessible for an implantation of basic sandwich set-ups by minimal invasive surgical techniques. Finally, according to the individual need, multiple basic sandwich set-ups could be stacked like bricks underneath the renal capsule (Figure 12).

Coming back to the actual situation, intense basic research, more advanced culture experiments and first steps of implantation with stem/progenitor cells into laboratory animals have to be performed in the near future. In this coherence, it is important to find a choice of biocompatible fleece materials [72], to analyze the right size of implants and to investigate an exact placement of sandwich set-ups containing stem/progenitor cells underneath the renal organ capsule (Figure 12). Most important, conclusive data concerning the involved developmental processes have to be elaborated so that the proceeding of regeneration can be controlled step by step. Thus, the aim is to promote physiological development, while pathophysiological formation is early recognized. It is imaginable that a variety of morphogenic drugs will be detected in future that can be applied to initiate and to promote the process of regeneration. These molecules could be integrated in a fleece fiber to act as a local drug delivery system. However, only detailed knowledge about tubule development will make it possible to find a successful therapeutic protocol for guiding a secure regeneration of renal tubules in future biomedicine.

## 7. Conclusions

The aim of the presented experiments was to collect basic data dealing with the regeneration of renal tubules. Stem/progenitor cells were isolated from the cortex of neonatal rabbit kidney and mounted between layers of polyester fleece to create an artificial interstitium. Perfusion culture was performed with chemically defined IMDM containing aldosterone (1 × 10^-7^ M) as tubulogenic factor. Series of experiments demonstrate that an artificial interstitium is most advantageous for the spatial development of tubules. Applying this innovative approach culture medium is flowing through the space between the fleece fibers. This way, unstirred layers of fluid are prevented, while exchange of nutrition including respiratory gas is promoted. Keeping stem/progenitor cells at the interface of an artificial polyester interstitium the influence of newly developed morphogenic drugs, innovative drug-delivery systems in combination with advanced biodegradable fleece materials can be tested. The challenging aim for the future is to implant stem/progenitor cells mounted within an artificial interstitium and to find a protocol for guiding the process of regeneration within a diseased kidney.
